# Distance of Residence From the Cancer Center Influences Perioperative Outcomes After Robotic-Assisted Pulmonary Lobectomy?

**DOI:** 10.7759/cureus.28646

**Published:** 2022-08-31

**Authors:** Shruti Kulkarni, Liwei Chen, Anastasia Jermihov, Frank O Velez, Carla C Moodie, Joseph R Garrett, Jacques P Fontaine, Eric M Toloza

**Affiliations:** 1 Medical Education, University of South Florida Health Morsani College of Medicine, Tampa, USA; 2 Surgery, University of South Florida Health Morsani College of Medicine, Tampa, USA; 3 Thoracic Oncology, Moffitt Cancer Center, Tampa, USA; 4 Surgery and Oncologic Sciences, University of South Florida Health Morsani College of Medicine, Tampa, USA

**Keywords:** pulmonary lobectomy, robotic surgery, perioperative outcomes, cancer center, distance

## Abstract

Introduction

Increased distance of residence from the hospital has been previously associated with worse postoperative outcomes, especially increased hospital length of stay (LOS) after elective surgery in the USA as well as after pulmonary lobectomy in Japan. We sought to determine if the distance from our cancer center affects postoperative outcomes after robotic-assisted pulmonary lobectomy.

Methods

We retrospectively analyzed 449 patients who underwent robotic-assisted pulmonary lobectomy by one surgeon for known or suspected lung cancer. Two patients were excluded due to incomplete data. Each patient’s residential ZIP code was used to determine the distance of their primary residence from our cancer center. Group 1 consisted of patients living less than 120 miles away while Group 2 consisted of patients living more than 120 miles away. Demographic factors, preoperative comorbidities, the incidence of postoperative complications, chest tube duration, and hospital LOS were compared by the Pearson chi-square or Kruskal-Wallis tests, and Kaplan-Meier survival was compared by Cox regression. Statistical significance was established as *p*≤0.05.

Results

Group 1 was found to have a higher mean body mass index (BMI) (28.3 kg/m^2^) than Group 2 (27.0 kg/m^2^; *p*=.031). Group 1 also tended to have a higher rate of preoperative hypertension (HTN; 59%) than Group 2 (47%; *p*=.018). No other preoperative comorbidities were significant. Median hospital LOS was found to differ between Group 1 (4 days) and Group 2 (5 days; *p*=.048). Postoperative complication rates did not differ between Group 1 (35%) and Group 2 (40%; *p*=.370). Median chest tube durations for Group 1 (4 days) vs. Group 2 (4 days) did not differ (*p*=.093). Five-year overall survival (OS) did not differ between the two groups (*p*=.550).

Conclusions

Longer distance from patient residence to our cancer center was associated with higher BMI, higher rates of preoperative HTN, and longer LOS. Postoperative complication rates, chest tube duration, and five-year OS were not significantly affected by distance. These results supported similar results in a Japanese study that indicated distance extends the LOS, regardless of the type of transportation used by patients. Further research analyzing the effects of socioeconomic status and insurance coverage on perioperative outcomes should be conducted to identify subpopulations in the USA that suffer disparities in access to and delivery of healthcare.

## Introduction

In the United States, lung cancer is the second most diagnosed cancer and remains the leading cause of cancer mortality despite significant advances in healthcare [1-2]. Worldwide, lung cancer is in the top three most commonly diagnosed cancers, being the number one type of cancer for both incidence and mortality in men [3]. Generally, surgical resection is the treatment of choice in patients with early-stage cancer [4]. Surgical lung resection, particularly lobectomy, whether via thoracotomy, video-assisted thoracoscopic (VATS), or robotic-assisted video-thoracoscopic (RAVT) approach, is the generally accepted treatment option for early-stage lung cancers. Compared to lobectomy via thoracotomy, VATS lobectomy has lower rates of postoperative complications and shorter hospital LOS [5]. Further, RAVT lobectomy has been shown to facilitate lung resection by providing a computer-assisted platform with wristed instrumentation as well as the elimination of hand tremors [6].

 While the effects of demographics, comorbidities, and cancer stage have been investigated on rates of postoperative complications and hospital length of stay (LOS), there have been few studies conducted investigating the relationship between distance traveled from residence to hospital and subsequent perioperative outcomes, such as hospital LOS, after surgical procedures for cancer. One study conducted on National Cancer Institute (NCI)-designated institutions found that increased travel time to the cancer center was associated with decreased utilization of the center [7]. The same researchers also noted that the median travel time in the South to an NCI-designated institution was 160 minutes [8]. Patients undergoing other surgical treatments across the United States were also found to have decreased utilization of cancer centers as the distance from the hospital increased [9-10]. However, no analysis was done of the amount of time spent during each admission for the patients who live furthest away. In other studies, patients undergoing elective pancreatic resection have been shown to have prolonged LOS when they came from further away from the hospital [11]. This helped develop the theory that patients who travel from further away may have longer admission LOS than those who live near the center. This study aims to investigate the relationship between distance traveled to our cancer center and perioperative outcomes after RAVT lobectomy in an effort to better understand barriers to discharge from the hospital to these patients.

This study was presented in part at the 15th Annual Academic Surgical Congress in Orlando, FL (USA), on February 4, 2020.

## Materials and methods

We retrospectively reviewed consecutive patients who underwent robotic-assisted pulmonary lobectomy by one surgeon from September 2010 through August 2018 at a single NCI-designated cancer center located in a suburban area in Tampa, FL, USA. This database protocol was approved by our institution’s Scientific Review Committee and our university’s Institutional Review Board, which waived informed consent for this retrospective study, which is considered a review of existing data. Additionally, the patients reviewed for the study all gave informed consent for fiberoptic bronchoscopy, RAVT wedge resection and/or RAVT (completion) lobectomy, mediastinal lymph node dissection (MLND), and possible thoracotomy. Some patients also gave informed consent for any anticipated en bloc chest wall and/or vertebral resection, with possible chest wall and/or vertebral reconstruction. Through our institutional surgical informed consent, patients also gave permission to use surgery-related and tissue-related data for education and research purposes.

All our patients underwent fiberoptic bronchoscopy by the operating surgeon after the induction of general anesthesia. After placement of the dual-lumen endotracheal tube, the patient is placed in either the right or left lateral decubitus position. Our robotic-assisted lobectomy technique utilizes a three-port system, which includes a 4-cm camera port along the sixth intercostal space (ICS) at the anterior axillary line, which doubles as the assistant’s access port, and two 1-cm instrument ports along the third ICS at the anterior axillary line and along the ninth ICS at the posterior axillary line.

From September 2010 through December 2011, our group used the daVinci® “S”™ robotic surgical system (Intuitive Surgical, Sunnyvale, California) with the “Si”™ system being used from January 2012 to March 2017 and the “Xi”™ system being used from April 2017 to the present. Lobectomy is performed with the pulmonary vein divided first, then the division of the pulmonary artery branch(es) and bronchus, and then the completion of the pulmonary fissures. After delivery of the lobectomy within an endopouch through the sixth ICS port incision, robotic-assisted complete MLND is then performed. At the end of the procedure, a 32-French chest tube is introduced through the 9th ICS port incision and connected to drainage at −20 cmH2O continuous suction.

We utilized the patients’ residential ZIP codes as provided in their electronic medical records (EMR) as an approximation for their residential location. Each ZIP code was entered into Google Maps, and the distance from that ZIP code to our cancer center was calculated. The distances were divided into two groups: residential ZIP codes less than or equal to 120 miles (<193 km) (Group 1) or more than 120 miles (>193 km) (Group 2) from our cancer center. These groups were chosen based on both previous studies and anecdotal evidence. Studies have shown multiple different median travel time points, ranging from 90 minutes one-way to 160 minutes to arrive at an NCI-designated institution [8,12]. Additionally, the same study revealed an average one-way travel time of 120-140 minutes for patients living in non-metropolitan areas. An average of these numbers was taken to approximately 120 miles (193 km) (estimating 120 minutes on a large motorway) to obtain our two groups.

Variables analyzed in this study included age, gender, body mass index (BMI), and preoperative comorbidities. Preoperative comorbidities included hypertension (HTN), hyperlipidemia (HLD), atrial fibrillation (AFib), diabetes mellitus (DM), chronic obstructive pulmonary disease (COPD), and previous cancer history, among others. Patients’ smoking status was also included in the analysis. Patients who had quit smoking within three months prior to surgery and those who were actively smoking at the time of surgery were considered current smokers. Those who had quit more than three months prior to surgery were considered former smokers. The cancer stage was also compared between the two study groups.

The primary outcome analyzed in this study was hospital LOS after RAVT lobectomy. Other outcomes analyzed included postoperative complications, chest tube duration, and in-hospital mortality.

We reported the mean and standard error of the mean (SEM) or median and interquartile range (IQR) for continuous variables and frequency (percentage) for categorical variables. Hospital LOS was reported as median ± IQR. Differences in continuous variables between longer-distance and shorter-distance groups were compared using the student’s t-test, Wilcoxon rank-sum test (two groups), or Kruskal-Wallis test. We used the chi-square test or Fisher’s exact test to investigate the association between categorical variables. The inverse Gaussian (V (μ) = μ3) regression model was utilized to evaluate predictive variables for hospital LOS while controlling for other covariates. Overall survival (OS) rates were assessed using Kaplan-Meier curves, and differences in survival curves were compared using log-rank tests with Bonferroni's correction. Statistical analyses were performed using SAS 9.4 (SAS Institute Inc., Cary, NC). A p-value of ≤0.05 was considered to indicate statistical significance.

## Results

Demographics and preoperative comorbidities

Of 449 consecutive patients who underwent RAVT lobectomy, two patients were excluded due to incomplete data. Our remaining study population of 447 patients included 189 (42.3%) men and 258 (57.7%) women (Table 1). The mean age at surgery was 67.5 years (range 24 to 87 years), with there being no significant difference in mean age between the two groups (67.7 vs 66.4; p=0.249). There were 94 patients (21.0%) living more than 120 miles (>193 km; Group 2) from our cancer center. Distance had a significant effect on the hospital LOS for patients, with a distance of >120 miles (>193 km) from the hospital increasing the LOS by 1.21 times that of patients in Group 2. Among baseline characteristics for patients, only BMI and preoperative HTN differed significantly between the two distance groups, with BMI higher in Group 1 (28.3 kg/m^2^ vs 27.0 kg/m^2^; p=0.031; Table 1) and preoperative HTN more common in Group 1 as well (59.2% vs. 46.8%; p=0.031, Table 2).

**Table 1:** Baseline Characteristics and Clinical Outcomes After Robotic-Assisted Pulmonary Lobectomy: Demographics *SEM = standard error of mean; BMI = body mass index; BSA = body surface area; Preop = preoperative; FEV1% = forced expiratory volume in 1 second as a percent of predicted; SES = socio-economic status; **p-values obtained by student's *t*-test to compare means and by Chi-square (or Fisher's exact) test to compare proportions (percentages)

Variables	Distance		
	≤120 miles (<193 km) (N = 353)	>120 miles (>193 km) (N = 94)	*p*-value**
Age, year; mean ± SEM*	67.7 ± 0.5	66.4 ± 1.1	0.249
BMI*, kg/m^2^; mean ± SEM	28.3 ± 0.3	27.0 ± 0.5	0.031
BSA*, m^2^; mean ± SEM	1.89 ± 0.01	1.87 ± 0.03	0.356
Preop* FEV1%*, mean ± SEM	87.7 ± 1.0	85.9 ± 2.3	0.432
SES* Below 3x Poverty, n (%)	38 (11.0%)	12 (13.0%)	0.589
Gender Male, n (%)	150 (42.5%)	39 (41.5%)	0.861
Smoking Status, n (%)	-	-	0.876
Current	113 (32.0%)	29 (30.9%)	-
Former	174 (49.3%)	49 (52.1%)	-
Never	66 (18.7%)	16 (17.0%)	-

**Table 2:** Baseline Characteristics and Clinical Outcomes after Robotic-Assisted Pulmonary Lobectomy: Preoperative Comorbidities *Preop = preoperative; CAD = coronary artery disease; MI = myocardial infarction; COPD = chronic obstructive pulmonary disease; GERD = gastroesophageal reflux disease; **p-values obtained by the chi-square (or Fisher's exact) test

Preop Variables, n (%)	Distance		*p*-value**
	≤120 miles (<193 km) (N = 353)	>120 miles (>193 km) (N = 94)	
CAD* or MI*	57 (16.2%)	15 (16.0%)	0.948
COPD*	73 (20.7%)	17 (18.15)	0.569
Cerebrovascular Accident	15 (4.3%)	3 (3.2%)	0.775
Heart Valve Disease or Cardiomyopathy	21 (6.0%)	9 (9.6%)	0.217
Atrial Fibrillation	24 (6.8%)	5 (5.3%)	0.596
Other Arrhythmias	16 (4.6%)	3 (3.2%)	0.776
Carotid Stenosis	17 (4.8%)	5 (5.3%)	0.792
Congestive Heart Failure	7 (2.0%)	1 (1.1%)	1.000
Hypertension	209 (59.2%)	44 (46.8%)	0.031
Hyperlipidemia	175 (49.7%)	38 (40.4%)	0.109
Peripheral Vascular Disease	13 (3.7%)	4 (4.3%)	0.765
Obstructive Sleep Apnea	21 (6.0%)	10 (10.6%)	0.114
Asthma	29 (8.2%)	4 (4.3%)	0.190
Pneumonia	35 (9.9%)	4 (4.3%)	0.083
Pulmonary Fibrosis	4 (1.1%)	1 (1.1%)	1.000
Pulmonary Embolism	14 (4.0%)	5 (5.3%)	0.568
Cirrhosis or Liver failure	2 (0.6%)	0 (0.0%)	1.000
Pancreatitis	3 (0.9%)	3 (3.2%)	0.111
GERD*	75 (21.3%)	14 (14.9%)	0.171
Kidney Disease	12 (3.4%)	3 (3.2%)	1.000
Chronic Anemia	8 (2.3%)	2 (2.1%)	1.000
Coagulation, Hemophilias, or Thrombocytopenia	3 (0.9%)	3 (3.2%)	0.111
Diabetes Mellitus	65 (18.4%)	11 (11.7%)	0.124
Previous Cancer	155 (43.9%)	37 (39.4%)	0.429
Chemotherapy	9 (2.8%)	6 (6.8%)	0.103

Postoperative complication rates did not differ between the two groups, whether overall or individual complications (Table 3). Hospital LOS differed between the two groups significantly (Table 4). Group 1 was found to have a median LOS of 4 days while Group 2 had a median LOS of five days (p=0.046).

**Table 3:** Baseline Characteristics and Clinical Outcomes After Robotic-Assisted Pulmonary Lobectomy: Postoperative Complications **p-values obtained by the chi-square (or Fisher's exact) test

Complication Variables	Total n = 447	Distance ≤120 miles (<193 km) (n = 353)	Distance >120 miles (>193 km) (n = 94)	*p*-value**
Overall postoperative complications	163 (36.5%)	125 (35.4%)	38 (40.4%)	0.369
Pulmonary-related complications	-	-	-	-
Prolonged air leak for >5 days	94 (21.0%)	71 (20.1%)	23 (24.5%)	0.357
Prolong air leak for >7 days with or without subcutaneous emphysema	85 (19.0%)	65 (18.4%)	20 (21.3%)	0.530
Pneumonia	28 (6.3%)	23 (6.5%)	5 (5.3%)	0.671
Chyle leak	18 (4.0%)	12 (3.4%)	6 (6.4%)	0.233
Mucous plug requiring intervention	17 (3.8%)	12 (3.4%)	5 (5.3%)	0.371
Respiratory failure	8 (1.8%)	6 (1.7%)	2 (2.1%)	0.677
Hypoxia	5 (1.1%)	4 (1.1%)	1 (1.1%)	1.000
Hemothorax	5 (1.1%0	5 (1.5%)	0 (0.0%)	0.589
Pneumothorax s/p CT removal	8 (1.8%)	7 (2.0%)	1 (1.1%)	1.000
Aspiration	6 (1.3%)	6 (1.7%)	0 (0.0%)	0.351
Effusion/ empyema	1 (0.2%)	1 (0.3%)	0 (0.0%)	1.000
Pleural effusion	2 (0.5%)	2 (0.6%)	0 (0.0%)	1.000
Cardiovascular-related complications	-	-	-	-
Atrial fibrillation	48 (10.7%)	40 (11.3%)	8 (8.5%)	0.433
Other arrhythmia requiring intervention	6 (1.3%)	6 (1.7%)	0 (0.0%)	0.351
Shock/multi-organ system failure (MOSF)	5 (1.1%)	5 (1.4%)	0 (0.0%)	0.589
Cardiovascular arrest	3 (0.7%)	2 (0.6%)	1 (1.1%)	0.508
Myocardial infarction (MI)	2 (0.5%)	2 (0.6%)	0 (0.0%)	1.000
Cerebrovascular accident (CVA)	1 (0.2%)	0 (0.0%)	1 (1.1%)	0.210

**Table 4:** Baseline Characteristics and Clinical Outcomes After Robotic-Assisted Pulmonary Lobectomy: Conversion, Pathologic Stage, Perioperative Outcomes *SEM = standard error of mean; IQR = interquartile range; Postop = postoperative; LOS = length of stay; **p-values were obtained by the student's *t*-test of means, Kruskal-Wallis test of medians, or chi-square (or Fisher's exact) test of proportions (percentages)

Variables	Distance		
	≤120 miles (<193 km) (N = 353)	>120 miles (>193 km) (N = 94)	*p*-value**
Tumor size, cm; mean ± SEM	3.2 ± 0.1	3.5 ± 0.2	0.125
Pathologic Stage	-	-	0.338
1	184 (57.0%)	40 (46.0%)	-
2	66 (20.4%)	22 (25.3%)	-
3	65 (20.1%)	22 (25.3%)	-
4	8 (2.5%)	3 (3.5%)	-
Skin-to-Skin Operative Time, min; median (IQR*)	176 (146-220)	184 (152-239)	0.140
Total Operative Time, min; median (IQR)	214 (183-258)	236 (187-290)	0.113
Estimated Blood Loss, mL; median (IQR)	150 (100-250)	200 (100-300)	0.095
Conversion to Thoracotomy, n (%)	17 (4.8%)	9 (9.6%)	0.080
Postop* Complication, n (%)	125 (35.4%)	38 (23.3%)	0.369
Chest Tube Duration, days; median (IQR)	4 (2-6)	4 (3-8)	0.055
Hospital LOS*, days; median (IQR)	4 (3-7)	5 (4-8)	0.046
In-Hospital Mortality, n (%)	4 (1.1%)	2 (2.1%)	0.610

Hospital LOS also correlated with age and preoperative forced expiratory volume in one second as percent of predicted (FEV1%), although the correlation between hospital LOS and tumor size did not reach significance (p=0.0735) (Table 5). Every 1 unit increase in FEV1% was correlated to a decrease in LOS of 15%. In addition, there were significant differences in hospital LOS for socioeconomic status (SES), gender, smoking status, preoperative CVA, preoperative COPD, preoperative HTN, preoperative pancreatitis, preoperative chronic anemia, and preoperative chemotherapy between the two groups (Table 6). An SES below 300% of the poverty line was associated with a longer LOS (5 d) than one above 300% of the poverty line (4 d; p=0.034). Male patients had a LOS that was 1.19 times as long as that of a female (5 d vs 4 d; p=0.004). Patients with any history of smoking (current or former smokers) had a one-day longer median LOS than never-smokers (4 d; p=0.001). Those with preoperative CVA had a longer LOS of 7 d compared to 4 d in those without this comorbidity (p=0.003). The same held true for those with preoperative COPD versus those without COPD (6 d vs 4 d; p=<0.001), for those with preoperative HTN versus those without (5 d vs 4 d; p=0.040), and for those with preoperative pancreatitis versus those without (9.5 d vs 4 d; p=0.045). Patients who had preoperative chronic anemia had a slightly shorter LOS (3.5 d) than those without (4 d; p=0.040). This was a decrease in mean LOS of 51%. Finally, those who had undergone preoperative chemotherapy were found to have a longer LOS (6 d) than those without (4 d; p=0.02), which was a 1.75-fold increase in LOS. Hospital LOS was also associated with conversion to thoracotomy (Table 6), with patients having been converted to thoracotomy having a LOS of 6 d vs 4 d for those without conversion (p=0.008), which is a 1.45-fold increase in LOS.

**Table 5:** Correlation Coefficients for Non-Distance Variables *LOS = length of stay; BMI = body mass index; Preop fEV1% = preoperative forced expiratory volume in 1 second as percent of predicted

		LOS (days)	Age	BMI	Preop FEV1%	Size of Tumor
Coefficient	LOS (days)*	1	0.1046	-0.0444	-0.166	0.0848
p-value	LOS (days)	-	0.0271	0.3493	0.0005	0.0735
N	-	447	447	447	443	446
Coefficient	Age	-	1	-0.0477	0.0348	-0.0461
p-value	Age	-	-	0.3143	0.4654	0.3314
N	-	-	447	447	443	446
Coefficient	BMI*	-	-	1	-0.0864	-0.0921
p-value	BMI	-	-	-	0.0694	0.052
N	-	-	-	447	443	446
Coefficient	Preop FEV1%*	-	-	-	1	-0.1237
p-value	Preop FEV1%	-	-	-	-	0.0092
N	-	-	-	-	443	442
Coefficient	Size of Tumor	-	-	-	-	1
p-value	Size of Tumor	-	-	-	-	-
N	-	-	-	-	-	446

**Table 6:** Comparisons of Hospital LOS After Robotic-Assisted Pulmonary Lobectomy for Non-Distance Variables *LOS = hospital length of stay; IQR = inter-quartile range; SES = socioeconomic status; Preop = preoperative; COPD = chronic obstructive pulmonary disease; CVA = cerebrovascular accident; HTN = hypertension; **p-values obtained by non-parametric Wilcoxon test

Variables	Hospital LOS* (days)	
	Median (IQR*)	*p*-value**
SES*	-	0.034
Below 3x Poverty	5 (4 - 9)	-
Above 3x Poverty	4 (3 - 7)	-
Gender	-	0.004
Male	5 (4 - 8)	-
Female	4 (3 - 6)	-
Smoking Status	-	0.001
Current Smoker	5 (3 - 7)	-
Former Smoker	5 (3 - 8)	-
Never	4 (3 - 5)	-
Conversion to Thoracotomy	-	0.008
Yes	6 (4 - 8)	-
No	4 (3 - 7)	-
Preop* COPD*	-	<0.0001
Yes	6 (4 - 10)	-
No	4 (3 - 6)	-
Preop CVA*	-	0.003
Yes	7 (5 - 9)	-
No	4 (3 - 7)	-
Preop HTN*	-	0.040
Yes	5 (3 - 8)	-
No	4 (3 - 6)	-
Preop Pancreatitis	-	0.045
Yes	9.5 (4 - 12)	-
No	4 (3 - 7)	-
Preop Chronic Anemia	-	0.040
Yes	3.5 (2 - 5)	-
No	4 (3 - 7)	-
Preop Chemotherapy	-	0.020
Yes	6 (4 - 14)	-
No	4 (3 - 7)	-

Multivariable analysis 

The inverse Gaussian multivariable regression model was used to determine the independent predictive variables for hospital LOS (Table 7). This multivariable model revealed that distance, male gender, preoperative FEV1%, preoperative COPD, preoperative chronic anemia, and preoperative chemotherapy were independent predictors for hospital LOS. The results showed that distance was an independent predictive variable for hospital LOS (p=0.008) while controlling for other covariates. Mean hospital LOS for patients living more than 120 miles was 1.23 times as long as that for patients living less than 120 miles from the cancer center.

**Table 7:** Inverse Gaussian Multivariable Regression Model for Hospital Length of Stay *CI = confidence interval; SES = socioeconomic status; Preop = preoperative; FEV1% = forced expiratory volume in one second as percent of predicted; CVA = cerebrovascular accident; COPD = chronic obstructive pulmonary disease

Variables	Mean Estimate of Regression Coefficient	95% CI* Lower Limit	95% CI Upper Limit	Wald Chi-Square	p-Value
Intercept	3.6	1.4	1.8	13.51	0.0002
Distance, >120 miles (>193 km)	1.21	1.05	1.41	6.62	0.0101
SES*, Below 3x poverty	1.19	0.97	1.46	2.88	0.0897
Gender, male	1.19	1.02	1.38	4.78	0.0288
Age	1.01	0.998	1.014	2.36	0.1242
Preop FEV1%*	0.85	0.77	0.95	7.78	0.0053
Current Smoker	1.19	0.995	1.433	3.65	0.0562
Past Smoker	0.98	0.85	1.13	6.17	0.7397
Conversion to thoracotomy	1.54	1.13	2.11	7.40	0.0065
Preop CVA*	1.16	0.83	1.63	0.79	0.3733
Preop Hypertension	1.10	0.98	1.24	2.58	0.1080
Preop COPD*	1.28	1.08	1.50	8.54	0.0035
Preop Pancreatitis	1.22	0.68	2.17	0.45	0.5019
Preop Chronic Anemia	0.49	0.36	0.66	21.64	<0.0001
Preop Chemotherapy	1.75	1.13	2.70	6.32	0.0119

**Figure 1:**
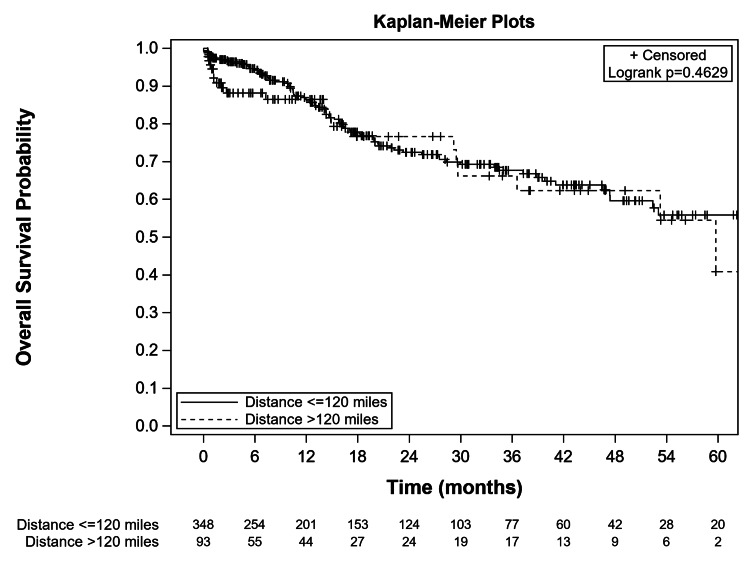
Kaplan-Meier Plot of 5-Year Overall Survival Curves for Patients Living ≤120 Miles (<193 km) versus >120 Miles (>193 km) from the Cancer Center

Five-year overall survival (OS) analysis showed that there was no difference between the two distance groups (p=0.463, Figure 1).

## Discussion

This study was primarily conducted to investigate the relationship between the distance of residence from the cancer center and hospital LOS after RAVT lobectomy. Multivariable regression modeling indicates that, when controlling for other covariates, distance was an independent predictive variable for longer hospital LOS after RAVT lobectomy. Distance has been shown to be associated with a longer hospital LOS in other elective procedures in the United States such as pancreatectomy, adenoma or cyst removal, and the Whipple procedure [11]. 

In the US, given that there is a lack of consistent public transportation, especially over long distances, the mode of transportation for the patient to return home must also be discussed and documented in discharge planning. While friends and family can often be relied upon to accept the burden of transporting a patient back home, this is not always the case for all patients. If a patient does not have access to a personal vehicle or cannot pay for gasoline or short-term accommodations, such as in a hotel or motel, they might struggle to find a reliable method of transportation back home, especially given the lack of good public transportation in much of this country. While the overall cost of hospitalization for one extra day is higher than that of a night in a hotel, the patient would not need to pay upfront for that extra day. Therefore, the immediate financial implications of prolonging the hospital stay would not be felt. This struggle might further prolong a patient’s LOS as they attempt to work through their situation prior to discharge. A study conducted in Japan determined that patients who can take advantage of good public transportation do not significantly differ in their hospital LOS regardless of the distance traveled [13].

Patients who are older also frequently have a longer hospital LOS after surgery, as do females [14-15]. Elderly patients also tend to have a higher rate of postoperative complications, most of which are pulmonary. These complications cause readmission in both pulmonary and non-pulmonary surgery. The most common reasons for an elderly patient to be readmitted after pulmonary lobectomy include pneumonia and pleural effusion [16]. These complications extend the patients’ hospital LOS regardless of where the patient was going after discharge. For patients who may be coming from a region without adequate follow-up care or for patients who have a long travel time to return home, the risk of potential post-discharge complications is a huge concern. It has been noted, however, that elderly patients undergoing surgical procedures do tend to leave the hospital for some sort of support, whether this is a rehabilitation facility or family. With that in mind, it is possible that our elderly patient population may have been discharged to a facility or nearby family after their procedure, thus allowing their hospital LOS to remain low.

When considering a patient’s disposition after surgery, while family and nearby rehabilitation facilities may be an option, SES might also play a role. Patients who reside in urban areas and/or who do not have private insurance are more frequently readmitted following VATS lobectomy than those who live in suburban areas [17]. Patients with public insurance (Medicare or Medicaid) often have worse postoperative outcomes after VATS lobectomy (more frequent readmissions after surgery, increased incidence of postoperative complications) than those with fully private insurance, with insurance type potentially serving as a surrogate for SES [18]. These patients also often require home oxygen after discharge, and some, especially those with poor preoperative performance status, are unable to return to their previous baseline [19]. For patients with a low SES background, having an extended hospital LOS can further delay their ability to get back home and get back to work by not being able to obtain necessary acute rehabilitation first. The lack of private insurance can also limit patients’ options for postoperative ambulatory care [20]. Patients with public insurance also tend to seek acute care more often than outpatient visits [20]. Traveling to and from the hospital can further increase that burden, especially since patients who travel long distances often come from lower SES areas [21]. Thus, relationships between a patient’s travel distance and SES should warrant further investigation.

The patient's smoking status prior to surgery was found to be a significant factor affecting postoperative hospital LOS. Surprisingly, former smokers were found to have a longer average hospital LOS than those who were current smokers. Patients who smoke, even those who are former smokers, tend to have a higher incidence of postoperative complications, such as atelectasis and pneumonia, than those who have never smoked [22]. Postsurgical quality of life, which includes frequency and level of dyspnea as well as return to physical baseline, was lower in patients who were current smokers at the time of surgery than those who had quit at any time prior to surgery [23]. While cigarette smoking causes a chronic inflammatory reaction in the alveoli, the level of pro-inflammatory cytokine release is significantly decreased, which may lead to decreased antibody response and a decreased ability of the patient to mount a defense against postoperative infections [24]. While the relationship between smoking status and postoperative complications was not directly studied in this paper and should be further explored, it is possible that an increased rate of postoperative complications in current or former smokers led to an extended hospital LOS.

This being a retrospective study dependent on the information provided by the patients’ EMR is a limitation of the study. The information provided was contingent on the patients being both accepting of providing their personal address as well as them providing an accurate ZIP code. In addition, no long-term follow-up was conducted. Since this study was conducted at a tertiary referral cancer center and with procedures performed by only one surgeon, it may not be readily extrapolated to the general public. A further limitation of this study is the inability to know from where the patients truly traveled when coming to the center. While the residential ZIP codes provided in the EMR were those of primary residences, many patients may live out-of-state for part of the year and stay near the cancer center on a seasonal basis, which may have confounded our results.

## Conclusions

It is important to consider the distance that a patient will have to travel to return home after a surgical procedure as well as to keep appointments for postoperative evaluation and subsequent follow-up. While discussions about discharge and disposition tend to happen within a day or two of the discharge date, it is imperative to begin thinking about the challenges that these patients might face earlier than usual. Pain control, complication occurrence, and the comfort of the patient with the travel home must all be discussed and incorporated into the plan for discharge.
